# Optical imaging of radiation-induced metabolic changes in radiation-sensitive and resistant cancer cells

**DOI:** 10.1117/1.JBO.22.6.060502

**Published:** 2017-06-16

**Authors:** Kinan Alhallak, Samir V. Jenkins, David E. Lee, Nicholas P. Greene, Kyle P. Quinn, Robert J. Griffin, Ruud P. M. Dings, Narasimhan Rajaram

**Affiliations:** a University of Arkansas, Department of Biomedical Engineering, Fayetteville, Arkansas, United States; b University of Arkansas for Medical Sciences, Division of Radiation Oncology, Little Rock, Arkansas, United States; c University of Arkansas, Department of Health, Human Performance, and Recreation, Fayetteville, Arkansas, United States

**Keywords:** optical redox ratio, radiation, metabolism

## Abstract

Radiation resistance remains a significant problem for cancer patients, especially due to the time required to definitively determine treatment outcome. For fractionated radiation therapy, nearly 7 to 8 weeks can elapse before a tumor is deemed to be radiation-resistant. We used the optical redox ratio of FAD/(FAD+NADH) to identify early metabolic changes in radiation-resistant lung cancer cells. These radiation-resistant human A549 lung cancer cells were developed by exposing the parental A549 cells to repeated doses of radiation (2 Gy). Although there were no significant differences in the optical redox ratio between the parental and resistant cell lines prior to radiation, there was a significant decrease in the optical redox ratio of the radiation-resistant cells 24 h after a single radiation exposure (p=0.01). This change in the redox ratio was indicative of increased catabolism of glucose in the resistant cells after radiation and was associated with significantly greater protein content of hypoxia-inducible factor 1 (HIF-1α), a key promoter of glycolytic metabolism. Our results demonstrate that the optical redox ratio could provide a rapid method of determining radiation resistance status based on early metabolic changes in cancer cells.

## 1 Introduction

Nearly 50% of all cancer patients each year are treated with radiation therapy, either alone or in combination with surgery or other forms of therapy.^1^ A critical challenge facing these patients is tumor resistance to radiation therapy. Currently, radiation response is determined based on tumor volume shrinkage using clinical imaging modalities 1 month after the treatment regimen. Because there is no present method to evaluate radiation response during the treatment period, patients who do not respond favorably are losing critical time when they could be switched to alternative forms of therapy, such as chemotherapeutics or surgery.

Conventional radiation therapy for solid tumors typically consists of fractionated dosing (2  Gy/day) for a period of 5 to 6 weeks. Fractionated therapy is believed to improve the treatment outcome by promoting reoxygenation and, hence, radiosensitizing previously hypoxic cancer cells.^2^ Reoxygenation due to fractionated radiotherapy is critically associated with treatment response.^3^^–^^5^ However, reoxygenation after radiation can also lead to the accumulation of tumor reactive oxygen species (ROS), which leads to the stabilization of hypoxia-inducible factor (HIF-1) even under well-oxygenated conditions^6^ and induces aerobic glycolysis.^7^ Aerobic glycolysis or increased glucose utilization under well oxygenated conditions has been implicated as a potential cause of radiation and chemotherapy resistance.^8^ The switch to aerobic glycolysis can promote radiation resistance in two possible ways: (1) utilization within the pentose phosphate shunt to maintain the NADPH-glutathione buffer and, hence, scavenge radiation-induced ROS,^9^ and (2) increased production of lactate, an important ROS scavenger, leading to decreased radiation sensitivity.^10^^,^^11^ Identifying such metabolic changes very early after commencing a radiation regimen would help identify patients with radiation-resistant tumors and allow them to avoid the toxic side effects of ineffective therapy.

Endogenous fluorescence from nicotinamide adenine dinucleotide (NADH) and flavin adenine dinucleotide (FAD) presents a label-free and validated^12^ method for quantifying cell metabolism.^13^ The optical redox ratio of FAD/(FAD+NADH) can be used to quantify the relative ratio of oxidative phosphorylation to catabolism.^12^ Specifically, increased oxidative phosphorylation is associated with an increase in the optical redox ratio due to oxidation of NADH to ${\mathrm{NAD}}^{+}$. Previous work has demonstrated a strong correlation between the optical redox ratio and the concentration ratio of ${\mathrm{NAD}}^{+}/({\mathrm{NAD}}^{+}+\mathrm{NADH})$ in various cell types.^12^^,^^14^ Decreases in the optical redox ratio have been associated with relative increases in catabolism, which can occur under hypoxic conditions^15^ or upon an increase in macromolecule synthesis (e.g., fatty acids).^12^ Furthermore, the optical redox ratio has been shown to be sensitive to a metabolic response to chemotherapy in tumor-derived organoids.^16^^,^^17^ Here, our objectives were to determine the early metabolic changes in cancer cells in response to radiation treatment and if these changes were associated with the level of observed radiation resistance. To accomplish our objective, we obtained an isogenic radiation-resistant cell line by repeated exposure of a parental, radiation-sensitive cell line to radiation. We measured the optical redox ratio at baseline (preradiation) and 24 h after exposure to a single, clinically relevant radiation dose of 2 Gy in the parental and resistant cell line, and found statistically significant differences between the cell lines. Our results suggest a potential increase in glycolytic metabolism in radiation-resistant cells after radiation. This information could be useful to predict and customize treatment for improved response rates.

## 2 Materials and Methods

### 2.1 Cell Culture, Irradiation, and Clonogenic Assay

**Fig. 1:**
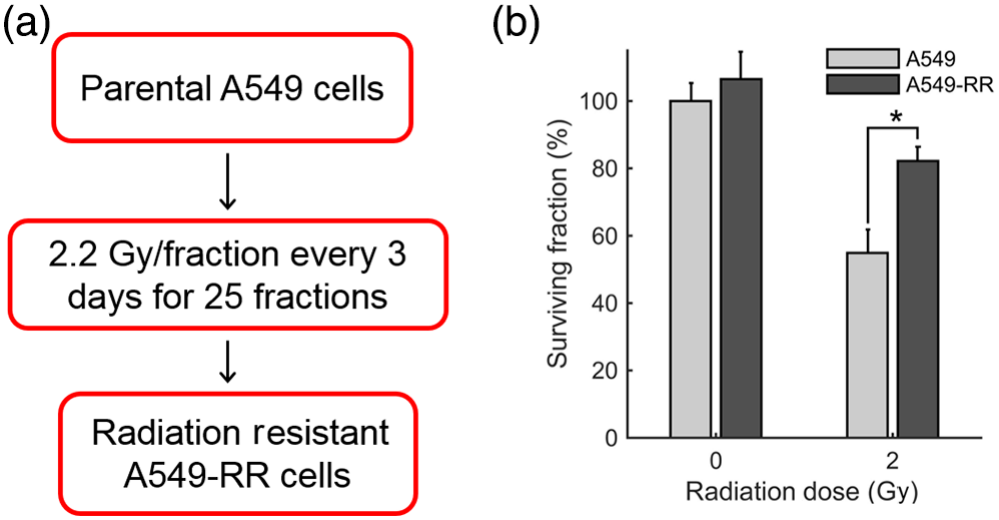
(a) Flowchart depicting the development of stable cultures of radiation-resistant A549 cells (A549-RR). (b) Clonogenic assay for survival of the A549-RR cells compared with the A549 cells when exposed to 2 Gy of radiation. Colonies (>50  cells) were quantified by fixing cells in methanol/acetic acid and staining with crystal violet. The number of identified colonies at 0 Gy divided by the seeding density was used to normalize the number of colonies formed per cell line at the various doses.

Human lung carcinoma A549 cells were purchased from American Type Culture Collection (ATCC; CCL185) and grown in Ham’s F-12K (Kaighn’s) medium mixed with 10% (v/v) fetal bovine serum and 1% (v/v) penicillin–streptomycin. These cells were irradiated at an average dose of 2.2 Gy every three days for a cumulative dose of 55 Gy (25 fractions) to create a radiation-resistant cell population [A549-RR; Fig. 1(a)]. An orthovoltage x-ray irradiator (X-Rad 320, Precision X-Ray, Inc., North Branford, Connecticut) was used for radiation. To assess clonogenic survival, cells in the exponential growth phase were trypsinized, washed, counted, and seeded at a concentration of 50 cells per well into 6-well plates. The plates were incubated overnight at 37°C and 5% ${\mathrm{CO}}_{2}$. Approximately 16 h after seeding, the flasks were treated with radiation and incubated for 8 days. To quantify colony formation, cells were washed with normal saline and fixed in 90:10 methanol:acetic acid for 30 min. Next, cells were stained for 2 h with 1% crystal violet in the methanol/acetic acid mixture. The number of resulting colonies (defined as consisting of >50  cells) per well was counted. The number of identified colonies at 0 Gy divided by the seeding density was used to normalize the number of colonies formed per cell line at the various doses, as described in our previous work.^18^^,^^19^ All experiments were performed in triplicates.

### 2.2 Two-Photon Imaging

We used a custom-built two-photon microscope, as described earlier,^20^ to collect endogenous fluorescence from NADH and FAD. The microscope consists of a MaiTai ultrafast Ti:Sapphire tunable laser source (Spectra-Physics, Santa Clara, California) that was tuned to 755 nm (NADH fluorescence; 20 mW) or 860 nm (FAD fluorescence; 40 mW). The laser powers at both wavelengths were carefully selected to avoid photobleaching. Images (512×512  pixels; 16-bit depth; 130  μm×130  μm; 200 frame average) were acquired using a resonant-galvo scanner operating at 30  frames/s via nondescanned GaAsP photomultipler tubes (H7422-40, Hamamatsu) with 460/40 nm (NADH) or 525/40 nm (FAD) bandpass filters, respectively, using a 20× water immersion objective (NA=0.75, working distance=0.51  mm). Cells were plated on glass slides at a density of 250,000  cells/well in a 6-well plate for 24 h. Preradiation measurements of NADH and FAD were acquired after 24 h by placing the glass slide in a heated chamber (37°C) perfused with 2 ml of regular growth media. A separate set of plates were radiated at a dose of 2 Gy using the orthovoltage x-ray irradiator and imaged 24 h later to obtain the postradiation fluorescence images. Redox images were created by calculated the pixel-wise ratios of FAD/(NADH+FAD) fluorescence and normalized using rhodamine measurements acquired at the end of each experiment.^20^ For statistical analysis and quantification, the average redox ratios of cell plates were calculated by separately computing the average FAD and NADH intensities from the respective images and taking the ratio of these values. For each cell line, three separate cell plates were imaged at each time point. Within each cell plate, three fields of view were acquired and six cells were randomly selected within each field of view. Two independent runs of these experiments were performed, and the data shown include both runs. Nested, two-way analysis of variance was used to determine statistical significant differences in the average redox ratio. The imaging time point and cell lines were considered fixed effects, whereas the cell plates nested within each group were considered random effects. Interactions between all effects were considered. *Post hoc* Tukey honest significant difference tests were used to evaluate statistical significance between specific cell groups.

### 2.3 Seahorse Metabolic Flux Assay

The protocol for the Seahorse metabolic flux assay was described in detail in a recent publication.^20^ Briefly, the Seahorse XFp analyzer uses oxygen and pH sensing dyes to determine oxygen consumption and proton production at baseline and in response to drugs that perturb mitochondrial activity. For this study, the radiation-resistant and sensitive cells were plated at a density of 10,000  cells/well. A separate 96-well plate with the same number of plated cells was used to determine cell count at the time of the experiment for the purpose of normalizing the data.^21^ Normalized oxygen consumption rate (n-OCR) was computed as the ratio of oxygen consumption rate (OCR) to proton production rate (PPR) to provide a relative measure of oxidative phosphorylation to glycolysis and to enable comparison to the optical redox ratio.

### 2.4 Immunoblotting

Protein extracts were homogenized and analyzed as previously described.^22^ Briefly, an assessment of HIF-1α protein content was accomplished using specific monoclonal antibody (NB100-105, Novus Biologicals, Littleton, Colorado) at a dilution of 1:1000 and appropriate horseradish peroxidase-linked secondary antibody alongside molecular weight ladder to verify size. Membranes were imaged on Protein Simple FluorChem M (Minneapolis, Minnesota) and analyzed using Image Studio Software (Li-Cor Biosciences, Lincoln, Nebraska). All blots were quantified within a linear range of exposure as previously optimized. All bands were normalized to the 45-kDa actin band of Ponceau S stain as a loading control. For each experiment, all groups were represented equally on each membrane and normalized to control condition.

## 3 Results and Discussion

Figure 1 shows the results of clonogenic survival assays conducted on both cell lines. The A549-RR cells showed a nearly 2.5-fold increase in survival after exposure to 2 Gy compared with the A549 parental cells. The enhancement in radiation resistance is highest at 2 Gy, the dose used to develop radiation resistance.

**Fig. 2:**
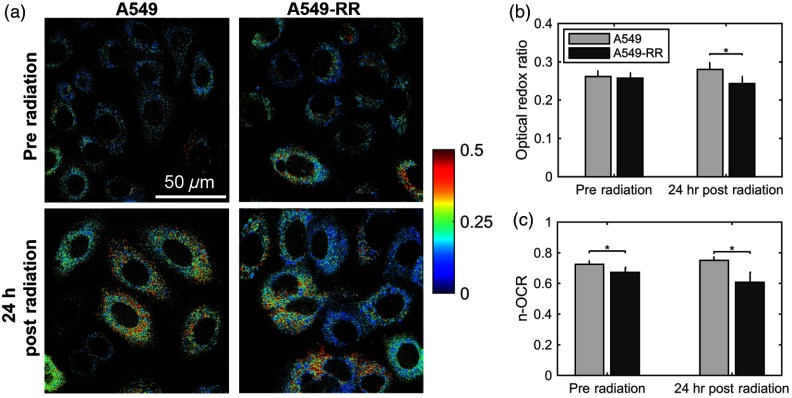
Radiation causes a decrease in the optical redox ratio after 24 h in the radiation-resistant cells, indicating increased glycolytic metabolism. (a) Representative redox images of parental and radiation-resistant A549 cells at baseline prior to radiation and 24 h after 2 Gy of radiation. (b) Quantification of redox ratio images indicates a statistically significant decrease in the optical redox ratio 24 h after radiation in the A549-RR cells compared with the parental A549 cells (p=0.01). (c) Differences in the n-OCR (calculated as OCR/PPR) are consistent with the optical redox ratio. Asterisks placed above bars indicate statistical significance. Error bars in panels (b) and (c) represent standard deviation of the mean plate value.

Figure 2(a) shows representative images of A549 and A549-RR cells at baseline and 24 h after exposure to 2 Gy of radiation. The average redox ratio was not significantly different between the two cell lines at baseline [Fig. 2(b)]. However, 24 h after radiation, there was a statistically significant (p=0.01) decrease in the redox ratio of A549-RR cells compared with the parental A549 cells. For both cell lines, the change in redox ratio postradiation was not significantly different compared with their respective baseline measurements. We did not observe any changes in cell confluency 24-h postradiation, and therefore, these measurements reflect radiation-induced metabolic changes. The change in optical redox ratio after radiation is consistent with measurements of the n-OCR, as determined by the Seahorse assay [Fig. 2(c)]. Additionally, n-OCR for the two cell lines was also significantly different at baseline (p<0.05). The lack of significant difference in the optical redox ratio between the two cell lines at baseline may be related to differences in macromolecule biosynthesis or NADPH concentrations that can affect the redox ratio^12^ but would not be reflected in the n-OCR. These results indicate that the A549-RR cells have decreased levels of oxygen consumption both at baseline and postradiation and resort to increased glycolysis after radiation to potentially reduce ROS-induced toxicity. Increased glucose catabolism under normoxic, well-oxygenated conditions is representative of an aerobic glycolysis phenotype^7^ and is consistent with a previous study that demonstrated elevated glucose uptake and decreased oxygen consumption in radiation-resistant head and neck cancer cells.^9^

**Fig. 3:**
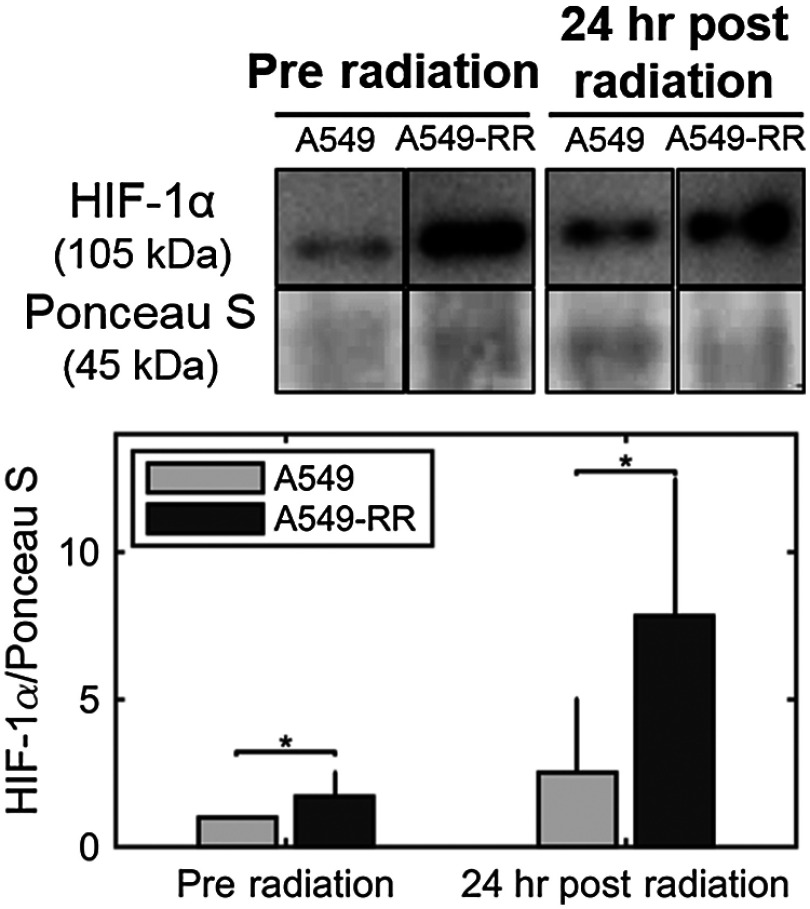
Radiation causes a significant increase in HIF-1α in the radiation-resistant cells 24 h after radiation. Western blots of HIF-1α protein expression demonstrate statistically significant differences between A549 and A549-RR cells at baseline and 24 h after radiation, indicating reoxygenation-induced HIF-1α expression in the A549-RR cells. Representative images are from the same gel and the same image. The 45-kDa actin band in the Ponceau S stain was used as a loading control.

To determine whether the shift to increased glycolysis was associated with upregulation of HIF-1, we determined the protein content of HIF-1α at baseline and 24 h after exposure to radiation. HIF-1 is a master regulator of oxygen homeostasis and directly targets several genes involved in glucose metabolism.^23^ HIF-1α protein content in the A549-RR cells was significantly greater after radiation compared to baseline (p=0.004) and compared with parental A549 cells after radiation (p=0.01; Fig. 3). Taken together with results from Fig. 2, these data point to a possible role for rapid activation of HIF-1α in decreasing oxygen consumption and increasing glycolytic metabolism in the A549-RR cells under normoxic conditions. Further studies of the optical redox ratio in these cell lines using HIF-1 inhibitors will elucidate a causal role for HIF-1α in promoting these metabolic changes.

In summary, we have presented the optical redox ratio as a potential noninvasive method for identifying early metabolic changes in cancer cells that may be indicative of radiation resistance. The matched model used here provides an excellent model to study molecular and metabolic pathways associated with radiation resistance in isogenic cell lines. The metabolic changes identified here using endogenous fluorescence are consistent with a recent study by Campos et al.^24^ that showed significantly lower mean NADH lifetime in UM-SCC-22B head and neck cancer cells exposed to 10 Gy radiation compared with normal oral keratinocytes, with a lower lifetime indicative of increased glycolysis. They also identified a significant increase in HIF-1α expression in the UM-SCC-22B head and neck cancer cells as early as 30 min after radiation.^24^ Other studies using models of radiation resistance have also identified increased HIF-1α expression in radiation-resistant cells^25^ and increased uptake of glucose that is shunted through the pentose phosphate pathway.^9^ Furthermore, radiation-resistant cells were also found to have significantly higher levels of fatty acid synthesis,^9^ which is interesting given that an increase in fatty acid synthesis has been shown to result in a lower redox ratio.^12^ In addition to glucose, glutamine has also been identified as a possible source of synthesis of glutathione, an ROS scavenger that helps inhibit radiosensitivity.^26^ We plan to extend our studies to include earlier time points, beginning immediately (within minutes) after radiation and determine the specific pathways of glucose utilization in the radiation-resistant cells. These investigations could potentially guide the selection of optimal time points for measuring the optical redox ratio in patient biopsy-derived organoids to provide a rapid determination of tumor radiation resistance and guide treatment planning.

## References

[1] Delaney G. et al., “The role of radiotherapy in cancer treatment,” Cancer 104(6), 1129–1137 (2005). CANCAR 0008-543X 10.1002/(ISSN)1097-0142 16080176

[2] Fowler J., “The rationale of dose fractionation,” in The Relationship of Time and Dose in the Radiation Therapy of Cancer, pp. 6–23, Karger Publishers (1969).

[3] Dietz A. et al., “Prognostic impact of reoxygenation in advanced cancer of the head and neck during the initial course of chemoradiation or radiotherapy alone,” Head Neck 25(1), 50–58 (2003). 10.1002/(ISSN)1097-0347 12478544

[r4] Grau C. Overgaard J., “The influence of radiation dose on the magnitude and kinetics of reoxygenation in a C3H mammary carcinoma,” Radiat. Res. 122(3), 309–315 (1990). RAREAE 0033-7587 10.2307/3577761 2356285

[5] Olive P. L., “Radiation-induced reoxygenation in the SCCVII murine tumour: evidence for a decrease in oxygen consumption and an increase in tumour perfusion,” Radiother. Oncol. 32(1), 37–46 (1994). RAONDT 0167-8140 10.1016/0167-8140(94)90447-2 7938677

[6] Moeller B. J. et al., “Radiation activates HIF-1 to regulate vascular radiosensitivity in tumors: role of reoxygenation, free radicals, and stress granules,” Cancer Cell 5(5), 429–441 (2004). 10.1016/S1535-6108(04)00115-1 15144951

[7] Zhong J. et al., “Radiation induces aerobic glycolysis through reactive oxygen species,” Radiother. Oncol. 106(3), 390–396 (2013). RAONDT 0167-8140 10.1016/j.radonc.2013.02.013 23541363 PMC3770265

[8] Pitroda S. P. et al., “STAT1-dependent expression of energy metabolic pathways links tumour growth and radioresistance to the Warburg effect,” BMC Medicine 7(1), 68 (2009). 10.1186/1741-7015-7-68 19891767 PMC2780454

[9] Mims J. et al., “Energy metabolism in a matched model of radiation resistance for head and neck squamous cell cancer,” Radiat. Res. 183(3), 291–304 (2015). RAREAE 0033-7587 10.1667/RR13828.1 25738895 PMC4465128

[10] Sattler U. G. et al., “Glycolytic metabolism and tumour response to fractionated irradiation,” Radiother. Oncol. 94(1), 102–109 (2010). RAONDT 0167-8140 10.1016/j.radonc.2009.11.007 20036432

[11] Quennet V. et al., “Tumor lactate content predicts for response to fractionated irradiation of human squamous cell carcinomas in nude mice,” Radiother. Oncol. 81(2), 130–135 (2006). RAONDT 0167-8140 10.1016/j.radonc.2006.08.012 16973228

[12] Quinn K. P. et al., “Quantitative metabolic imaging using endogenous fluorescence to detect stem cell differentiation,” Sci. Rep. 3(3432) (2013). SRCEC3 2045-2322 10.1038/srep03432 PMC3851884 24305550

[13] Chance B. et al., “Intracellular oxidation-reduction states *in vivo* the microfluorometry of pyridine nucleotide gives a continuous measurement of the oxidation state,” Science 137(3529), 499–508 (1962). SCIEAS 0036-8075 10.1126/science.137.3529.499 13878016

[14] Varone A. et al., “Endogenous two-photon fluorescence imaging elucidates metabolic changes related to enhanced glycolysis and glutamine consumption in precancerous epithelial tissues,” Cancer Res. 74(11), 3067–3075 (2014). 10.1158/0008-5472.CAN-13-2713 24686167 PMC4837452

[15] Mayevsky A. Chance B., “Intracellular oxidation-reduction state measured in situ by a multichannel fiber-optic surface fluorometer,” Science 217(4559), 537–540 (1982). SCIEAS 0036-8075 10.1126/science.7201167 7201167

[16] Shah A. T. et al., “Optical metabolic imaging of treatment response in human head and neck squamous cell carcinoma,” PLoS One 9(3), e90746 (2014). POLNCL 1932-6203 10.1371/journal.pone.0090746 24595244 PMC3942493

[17] Walsh A. J. et al., “Quantitative optical imaging of primary tumor organoid metabolism predicts drug response in breast cancer,” Cancer Res. 74(18), 5184–5194 (2014). 10.1158/0008-5472.CAN-14-0663 25100563 PMC4167558

[18] Dings R. P. et al., “Anginex synergizes with radiation therapy to inhibit tumor growth by radiosensitizing endothelial cells,” Int. J. Cancer 115(2), 312–319 (2005). IJCNAW 1097-0215 10.1002/(ISSN)1097-0215 15688384

[19] Dings R. P. et al., “Tumour thermotolerance, a physiological phenomenon involving vessel normalisation,” Int. J. Hyperthermia 27(1), 42–52 (2011). IJHYEQ 0265-6736 10.3109/02656736.2010.510495 21204622 PMC3086848

[20] Alhallak K. et al., “Optical redox ratio identifies metastatic potential-dependent changes in breast cancer cell metabolism,” Biomed. Opt. Express 7(11), 4364–4374 (2016). BOEICL 2156-7085 10.1364/BOE.7.004364 27895979 PMC5119579

[21] Simões R. V. et al., “Metabolic plasticity of metastatic breast cancer cells: adaptation to changes in the microenvironment,” Neoplasia 17(8), 671–684 (2015). 10.1016/j.neo.2015.08.005 26408259 PMC4674487

[22] Lee D. E. et al., “Translational machinery of mitochondrial mRNA is promoted by physical activity in Western diet-induced obese mice,” Acta Physiol. 218(3), 167–177 (2016). 10.1111/apha.2016.218.issue-3 27061106

[23] Semenza G. L., “HIF-1: upstream and downstream of cancer metabolism,” Curr. Opin. Genet. Dev. 20(1), 51–56 (2010). COGDET 0959-437X 10.1016/j.gde.2009.10.009 19942427 PMC2822127

[24] Campos D. et al., “Radiation promptly alters cancer live cell metabolic fluxes: an in vitro demonstration,” Radiat. Res. 185(5), 496–504 (2016). RAREAE 0033-7587 10.1667/RR14093.1 27128739 PMC4882764

[25] Zhao H. et al., “2-Methoxyestradiol enhances radiosensitivity in radioresistant melanoma MDA-MB-435R cells by regulating glycolysis via HIF-1α/PDK1 axis,” Int. J. Oncol. (2017). IJONES 1791-2423 10.3892/ijo.2017.3924 PMC5403226 28339028

[26] Sappington D. R. et al., “Glutamine drives glutathione synthesis and contributes to radiation sensitivity of A549 and H460 lung cancer cell lines,” Biochim. Biophys. Acta 1860(4), 836–843 (2016). BBACAQ 0006-3002 10.1016/j.bbagen.2016.01.021 26825773 PMC4768472

